# Height and Bone Mineral Density Are Associated with Naevus Count Supporting the Importance of Growth in Melanoma Susceptibility

**DOI:** 10.1371/journal.pone.0116863

**Published:** 2015-01-22

**Authors:** Simone Ribero, Daniel Glass, Abraham Aviv, Timothy David Spector, Veronique Bataille

**Affiliations:** 1 Department of Twin Research & Genetic Epidemiology, King’s College London, United Kingdom; 2 Section of Dermatology, Departments of Medical Sciences, University of Turin, Turin, Italy; 3 Dermatology Department, Northwick Park Hospital, Middlesex, United Kingdom; 4 Centre of Human Development and Ageing, University of Medicine and Dentistry of New Jersey, Newark, New Jersey, United States of America; 5 Dermatology Department, West Herts NHS Trust, Herts, United Kingdom; University of Connecticut Health Center, UNITED STATES

## Abstract

Naevus count is the strongest risk factor for melanoma. Body Mass Index (BMI) has been linked to melanoma risk. In this study, we investigate the link between naevus count and height, weight and bone mineral density (BMD) in the TwinsUK cohort (N = 2119). In addition we adjusted for leucocyte telomere length (LTL) as LTL is linked to both BMD and naevus count. Naevus count was positively associated with height (p = 0.001) but not with weight (p = 0.187) despite adjusting for age and twin relatedness. This suggests that the previously reported melanoma association with BMI may be explained by height alone. Further adjustment for LTL did not affect the significance of the association between height and naevus count so LTL does not fully explain these results. BMD was associated with naevus count at the spine (coeff 18.9, p = 0.01), hip (coeff = 18.9, p = 0.03) and forearm (coeff = 32.7, p = 0.06) despite adjusting for age, twin relatedness, weight, height and LTL. This large study in healthy individuals shows that growth via height, probably in early life, and bone mass are risk factors for melanoma via increased naevus count. The link between these two phenotypes may possibly be explained by telomere biology, differentiation genes from the neural crests but also other yet unknown factors which may influence both bones and melanocytes biology.

## Introduction

Having a large number of benign melanocytic naevi is one of the strongest risk factors for malignant melanoma [1]. Twin studies have demonstrated that 60% of the variation in naevi number between individuals is genetically determined [2–4]. Naevi typically involutes after the fourth decade of life in Caucasian populations and are rare in the elderly [3]. However, individuals who are susceptible to melanoma often have large numbers of naevi which persist until middle age or later [5]_._ This suggests that reduced senescence in the melanocytic system may be a good predictor of melanoma risk.

Telomere length is another important genetic marker of reduced senescence and melanoma risk, as both naevi count and melanoma are associated with longer telomeres [6–8]. Telomere length has been shown to have an impact on growth early in life especially in females [8]. Furthermore, increased bone density is associated with leukocyte telomere length (LTL) [9]. Despite a clear association between higher body mass index (BMI) and some cancers [10–14] the association with melanoma remains more controversial [15–18].

We therefore set out to examine at the relationships between naevus count, height, and weight and bone density (BMD) with adjustments for LTL to explore the role of reduced senescence and increased growth as a melanoma risk factor.

## Materials and Methods

### Twins UK

Twins were recruited from the TwinsUK adult twin registry. Caucasian monozygotic (MZ) and dizygotic (DZ) twin pairs were enrolled in the study between 1992 and 2003 to assess the heritability and genetics of age-related diseases (http://www.twinsUK.ac.uk). Members of TwinsUK have been shown to have similar disease and lifestyle characteristic to the general population and were not recruited on the basis of any specific trait or disease [19]. St. Thomas’ Hospital Research Ethics Committee approved the study, and all twins provided informed written consent. All the data is available upon request from the Twin Research Unit website: www.twinsuk.ac.uk/data-access/submission-procedure/.

### Bone mineral density

All twins have been recruited since 1992. At each visit a variety of clinical investigations were performed including bone mineral density (BMD) and weight measurement. Details of lifestyle, medical and drug history were obtained from comprehensive nurse-administered questionnaires. Using questionnaire data, twins who received Hormone Replacement Therapy or any osteoporosis treatment were excluded. BMD was measured at the lumbar spine (L1–4), total hip and forearm using dual energy X-ray absorptiometry (DEXA) Hologic QDR4500W (1996 to 2004). Daily quality control scans were performed using the spine phantom. All BMD measurements were performed using a standardized protocol of measurement [6–9]. Twins within each pair were always scanned at the same time. Intra-scanner reproducibility, expressed as a coefficient of variation from duplicate measurements in healthy volunteers one week apart, was 0.8% at the lumbar spine and 1.6% at the femoral neck. Height, weight and body surface area (BSA) was available on all twins.

### Skin data

Skin examination and data collection was undertaken on 3648 twins between January 1995 and December 2003. All twins underwent a skin examination including recording of skin type, hair and eye colour, freckles and mole count on 17 body sites performed by 5 trained nurses at St Thomas Hospital in London. This protocol for mole counting naevi has been published elsewhere and has been validated in other studies [3,20,21]. Skin type was assessed according to the Fitzpatrick classification.

### Telomere length

DNA was extracted from isolated leukocytes after an overnight fast and the mean leukocyte terminal restriction fragment length (TRFL) was measured using the Southern blot method as described previously [9]. Each DNA sample was resolved in duplicate (on different gels). If the difference between the duplicates was >5%, a third measurement was done and the mean of two results <5% apart was taken. This was only necessary in <5% of the assays. The coefficient of variation of the TRFL assay in this study was 0.92%. The laboratory conducting the TRFL measurements was blinded to all characteristics of the white cell donors.

### Data summary

3648 twins had complete data on naevi count, height weight and clinical data. For BMD and telomere length the data was restricted to 2119 subjects.

### Statistical Methods

We used X^2^ Pearson’s test and Student’s t–test to compare categorical and continuous variables, respectively. Kruskall-Wallis test was used to analyze the differences between median in non parametric analyses. Linear regression was used to calculate associations between naevus count, BMD, telomere and other possible confounding factors. Multicollinearity between variables was tested with the variance inflation factor without significant results.

Logistic regressions were carried out to look at the association between number of naevi and height. Number of naevi was categorized in groups of above or equal and below 50. Because of the non independence of twins, we controlled for familial aggregation by treating twin-pairs as clusters of information by using the robust regression cluster. Log likelihood ratio test was performed for models comparisons and to select variables which have to be included in the final models.

All statistical tests were two sided and p value ≤0.05 were considered significant. The analyses were performed in STATA 12 (StatCorp LP, College Station, TX, USA).

## Results

2119 female Caucasian twins with naevus count, height, weight and measurements for BMD at three sites (spine, forearm and hip) were included in this study. The mean age was 46.96±12.81 years (median 47.9 range 19–73 years). Regarding tanning ability, 12% had Fitzpatrick skin type I, 33% type II, 35% type III and 20% type IV. The mean total body naevus count was 34±41 (median 20-range 0 to 555). Naevus count was inversely correlated with age as expected at linear regression (coeff. −0.67±0.05, p<0.0001). Mean height was 162.44±6.14 cm (median 162, IQ range 158–166) and mean weight was 66.79±12.53 kg (median 64.7). BMD was inversely correlated with age for all three sites at linear regression (coeff. spine −21.16±1.46, coeff. forearm −62.47±3.78, coeff. hip −30.90±1.53, p<0.0001). BMD was positively associated with both height and weight at linear regression (height coeff. spine 9.47±0.71, coeff forearm 20.95 ±1.83, coeff. hip 9.52±0.78, p<0.0001; weight coeff. spine 26.88±1.45, coeff forearm 54.03±3.99, coeff. hip 35.55±1.64, p<0.0001).

**Figure 1:**
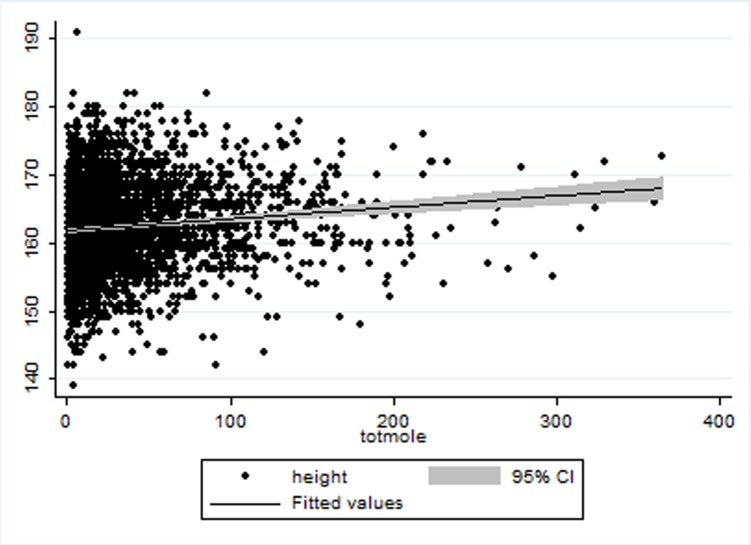
Scatter plot for height (Y axis) in association with naevus count (X axis).

Although BMI was positively associated with BMD at linear regression (coeff. spine 7.17±0.55, coeff. forearm 13.75±1.48, coeff. hip 10.51±0.63, p<0.0001), it was not associated with naevus count. When dissecting BMI, naevus count was positively associated with height at linear regression (coeff. 0.39±.013, p = 0.003) (Fig. 1) but not weight even after adjusting for twin relatedness, age and body surface area (BSA).

Height was associated with naevus count in a multivariate linear model with further adjustments for LTL. Therefore LTL does not explain fully the association observed between height and an excess of naevi. Naevus count was categorized in two groups (above or equal to 50 and below 50) for logistic regression evaluating the effect of increasing height on naevus count. Height is positively associated with increasing number of naevi even after adjustment age and twin relatedness. (Table 1).

**Table 1:** Logistic regression on risk factors for HNC (total naevus count equal or above 50) in relation to increasing height. (Robust regression) (n = 2119).

**HIGH NAEVUS COUNT (≥50)**		**OR**	**P value**	**95% IC**
**AGE**		0.97	<0.001	0.96–0.97
**HEIGHT (CM)**	<158	1		
	158–162	1.38	0.016	1.06–1.80
	162.1–166	1.32	0.038	1.01–1.73
	>166	1.67	<0.001	1.28–2.17

**Figure 2:**
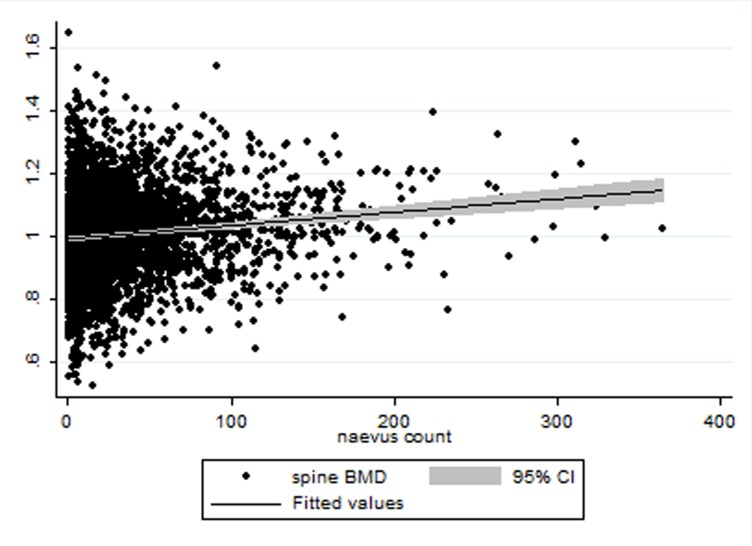
Scatter plot for Spine BMD (Y axis) referred to naevus count (X axis).

**Figure 3:**
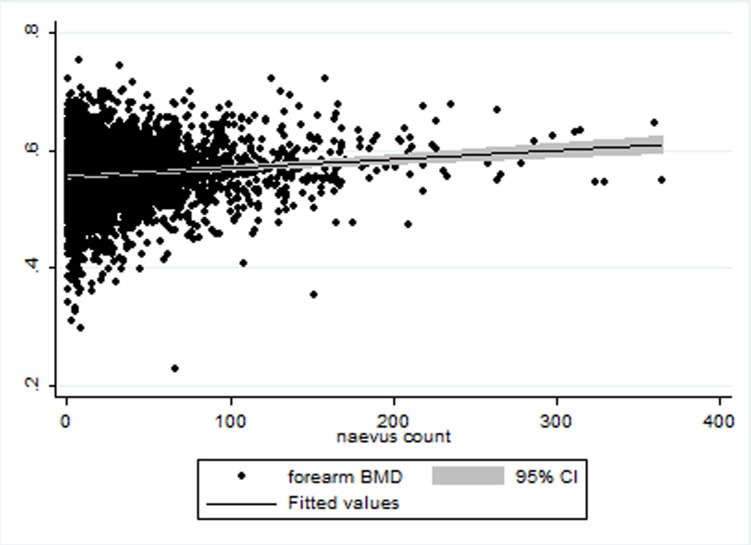
Scatter plot for Forearm BMD (Y axis) in association with naevus count (X axis).

**Figure 4:**
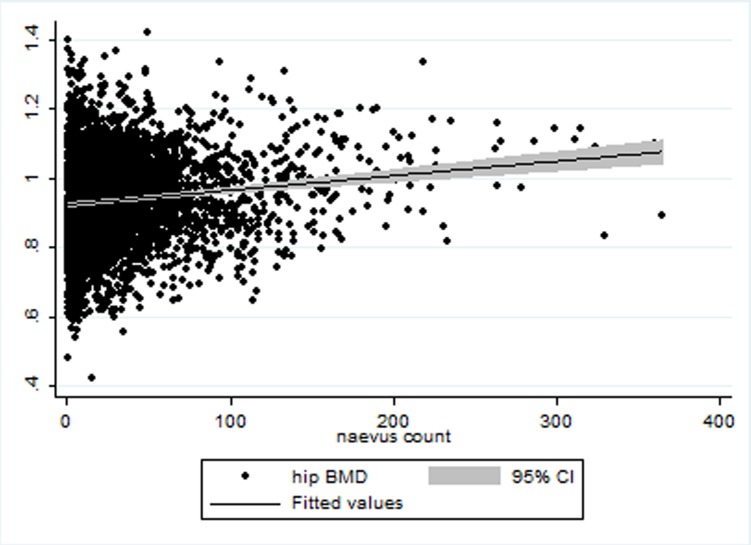
Scatter plot for hip BMD (Y axis) in association with naevus count (X axis).

BMD at the three sites was positively associated with total naevi count at univariate analysis of linear regression (coeff spine37.05±4.95, coeff forearm 84.43±11.95, coeff hip 42.39±5.38<0.001) (Figs. 2–4). BMD at the spine, forearm and hip was positively correlated with naevus count in linear regression analyses after adjusting for age, height, weight and twin relatedness: (spine coeff. 19.85399±5.2925, p<0.001; forearm coeff. 35.34±12.52, p = 0.03; hip coeff. 20.74619±6.12, p = 0.001). Further adjustments for telomere length did not affect the association between naevi counts and BMD for spine and hip, whilst for the forearm it was no longer significant (spine coeff 18.90, p = 0.01, hip coeff = 18.9 p = 0.03 and forearm coeff = 32.67, p = 0.06) (Tables 2, 3, 4). BMI and BSA were not included in the final model for the obvious risk of collinearity.

**Table 2:** Multivariate Linear Regression for the association between naevus count and spine BMD. (Robust regression) (n = 2119).

**NAEVUS COUNT**	**COEFF**	**P VALUE**	**95%CI**
**SPINE BMD**	18.90	0.013	3.94 33.86
**AGE**	−.43	0.000	−.59 −.26
**TELOMERE LENGHT**	3.82	0.005	.1.15 6.48
**HEIGHT**	.42	0.010	.10 .74
**WEIGHT**	.02	0.850	−.15 .18

**Table 3:** Multivariate Linear Regression for the association between naevus counts and hip BMD. (Robust regression) (n = 2119).

**NAEVUS COUNT**	**COEFF**	**P VALUE**	**95%CI**
**HIP BMD**	18.96	0.03	1.49 36.44
**AGE**	−.42	0.000	−.59 −.25
**TELOMERE LENGHT**	3.86	0.005	.1.17 6.55
**HEIGHT**	.44	0.16	.13 .77
**WEIGHT**	.004	0.961	−.17 .18

**Table 4:** Multivariate Linear Regression for the association between naevus count and forearm BMD (Robust regression) (n = 2119).

**NAEVUS COUNT**	**COEFF**	**P VALUE**	**95%CI**
**FOREARM BMD**	32.67	0.06	−2.14 67.48
**AGE**	−.44	0.000	−.61 −.28
**TELOMERE LENGHT**	3.77	0.005	1.11 6.44
**HEIGHT**	.44	0.008	.11 .76
**WEIGHT**	.04	0.619	−.12 .20

## Discussion

High number of naevi is the most powerful risk factor for melanoma and suggests a lack of senescence in the melanocytes in individuals at high risk [7–8]. We report here a positive correlation between the number of naevi and height after adjustment for age, weight and twin relatedness. Weight alone is not associated with naevi counts in our study and is not a predictor of melanoma risk.

The association between melanoma risk and BMI is controversial but previous studies have already showed a link with higher BMI [15–18,22]. In this study, we dissected BMI by looking at height and weight separately in relation to naevi count and showed that the association is maintained with height but not weight. However, a melanoma case control study looked at BMI, weight and height separately found an association between melanoma and weight but not height in contrast to our study [22].

The study reported here also explored the association between naevus count and BMD and showed a significant association at three sites. Spine BMD was the most significantly associated with naevus count and this could be explained by the fact that the spine is a trabecular bone and the rate of ageing is known to be greater in trabecular compared to cortical bones such as hip and forearm [7].

The association between height and BMD and high naevus count suggests that growth is important for melanoma rather than weight alone. Height has already been reported to be a risk factor for melanoma and other cancers [23–25]. Moreover height in childhood has already been linked to breast cancer risk so it is likely that factors affecting growth early in life are involved [26, 27]. The study reported here is looking at healthy individuals whom have not yet developed cancer. This study confirms an association between height and risk of melanoma using a powerful marker of risk phenotype for melanoma. Using naevus count allowed for the recruitment of a large number of individuals to explore these associations which would be difficult for melanoma. The only confounding factor which affected the significance of the association between BMD and naevi, apart from age, was height and LTL which suggests that it may be a “growth/reduced senescence phenotype” which links naevi, telomere length and BMD as risk factors for melanoma.

Though genetic make-up is a major determinant of height, modifiable early-life and pre-pubertal factors, e.g., childhood nutrition, illness, and socioeconomic status are also relevant so environmental factors may be at play as well [26]. Melanoma has already been associated with higher socio-economical status and nutrition may be one of the factors involved [28, 29].

Bone loss is also considered an index of ageing so our findings of higher BMD in subjects at risk of melanoma support the notion that the biological age of individuals with high naevus count might be younger than their chronological age, in line with their longer LTL [9]. Moreover, telomere biology is likely to play an important role in the BMD-naevi count association. However, despite adjusting for LTL, the association between naevi counts and BMD remained so LTL does not explain it all. Naevi involute with age; a process which is likely to be, in part, genetically driven [3–4]. However, the rate of at which naevi disappear with age varies greatly with some individuals still having a large number of naevi in late middle age. This phenomenon is more likely to be observed in families at high risk of melanoma [5]. Oncogene (BRAF and RAS) and tumour suppressor (p16, p14arf, p53, PTEN, Rb) driven cell senescence is important in melanocytes [30–32]. PTEN depletion and the pI3K pathway are also thought to be important in inhibiting BRAF induced senescence in naevi [33]. Telomere erosion as well genes in the p53 pathways also play a role in cell senescence [34–35]. However, examining markers of senescence does not differentiate benign naevi from melanoma so does not allow speculation of which factors influence progression from naevus to melanoma [36].

Similarly bone loss with age varies widely between individuals with levels of post-menopausal bone loss estimated between 0.5% and 3% per annum [37]. Fifty *per cent* of the variance in bone loss in postmenopausal Caucasian women is due to genetic factors [38]. Like naevus count, BMD has also been positively linked to LTL [16]. Interestingly, osteoblasts and melanoblasts originate from the neural crest [38, 39]. A complex network of genes and pathways are involved in cell differentiation and migration. Among these genes MITF, WNT/beta Catenin, SOX9, SOX2, PAX3, RANK/RANKL are important in melanocyte differentiation and melanoma [40,41]. Recent GWAS studies of osteoporosis have identified variants in many of the above genes as predictors of bone density [40–47]. There is therefore substantial evidence that many genes involved in bone metabolism are also crucial for melanocyte differentiation and invasion. Curves showing rates of bone loss and decrease in naevus count with age in this study are very similar supporting the hypothesis that factors determining the rate of ageing for these two phenotypes are likely to be linked.

This study shows an association between height, BMD and naevus count which are all influenced by LTL and supports the role of telomere biology in melanoma [6]. However, LTL does not explain the whole of these associations so other factors are at play. SNPs in the TERT gene and amplification in its promoter have been to linked to melanoma risk in recent studies [7,36,48–50]. The reduced senescence generated by longer LTL may have positive offsets in individuals at higher risk of melanoma with protection against osteoporosis and possibly other ageing phenotypes. Unfortunately, it is also likely to explain the observed increased risk of cancers (not only melanoma) in many families with an excess of naevi (especially in the presence of many atypical naevi). This shows the intricate balance between ageing and cancer as highlighted by Peeper *et al* [10]. Results of GWAS candidates for osteoporosis, BMI and telomere length are currently being investigated as possible candidates for naevi counts to discover new naevi/melanoma genes. However, recent GWAS for height and BMD are currently being reported with increasing sample size (as many as 250,000 individuals for height) showing very large numbers of SNPs variants for height (more than 600 SNPs to date) and BMD so the task is not easy. Genome-wide association studies have shown that many of the genes associated to height are linked to cancer pathways [51].

It is likely therefore that the connections reported here between naevus counts, height and BMD could be explained by a large network of genes involved in embryogenesis, development and growth which later may have an impact on cancer risk, especially genes involved in the common stem cell lineage shared by melanocytes and bone cells in the neural crest.
